# The role of astrocytes in Alzheimer’s disease: a bibliometric analysis

**DOI:** 10.3389/fnagi.2024.1481748

**Published:** 2024-11-27

**Authors:** Xiaoqiong An, Jun He, Bin Bi, Gang Wu, Jianwei Xu, Wenfeng Yu, Zhenkui Ren

**Affiliations:** ^1^Department of Laboratory Medicine, The Second People's Hospital of Guizhou Province, Guiyang, China; ^2^Key Laboratory of Molecular Biology, Guizhou Medical University, Guiyang, Guizhou, China; ^3^Key Laboratory of Human Brain Bank for Functions and Diseases of Department of Education of Guizhou Province, Guizhou Medical University, Guiyang, Guizhou, China; ^4^Guizhou Provincial Center for Clinical Laboratory, Guiyang, China; ^5^Center for Tissue Engineering and Stem Cell Research, Guizhou Medical University, Guiyang, China; ^6^Psychosomatic Department, The Second People's Hospital of Guizhou Province, Guiyang, China; ^7^Department of Pharmacology, School of Basic Medicine, Guizhou Medical University, Guiyang, China

**Keywords:** Alzheimer’s disease, astrocytes, bibliometrix, CiteSpace, VOSviewer

## Abstract

**Background:**

Alzheimer’s disease (AD) is a neurodegenerative disorder marked by cognitive decline and memory loss. Recent research underscores the crucial role of astrocytes in AD. This study reviews research trends and contributions on astrocytes in AD from 2000 to 2024, shedding light on the evolving research landscape.

**Methods:**

We conducted a bibliometric analysis using data from the Web of Science Core Collection, covering publications from January 1, 2000, to July 6, 2024, on “Alzheimer’s disease” and “astrocytes.” We identified 5,252 relevant English articles and reviews. For data visualization and analysis, we used VOSviewer, CiteSpace, and the R package “bibliometrix,” examining collaboration networks, co-citation networks, keyword co-occurrence, and thematic evolution.

**Results:**

Between 2000 and 2024, 5,252 publications were identified, including 4,125 original research articles and 1,127 review articles. Publications increased significantly after 2016. The United States had the most contributions (1,468), followed by China (836). Major institutions were the University of California system (517) and Harvard University (402). The Journal of Alzheimer’s Disease published the most articles (215). Verkhratsky A was the top author with 51 papers and 1,585 co-citations.

**Conclusion:**

Our extensive bibliometric analysis indicates a significant increase in research on astrocytes in AD over the past 20 years. This study emphasizes the growing acknowledgment of astrocytes’ crucial role in AD pathogenesis and points to future research on their mechanisms and therapeutic potential.

## Introduction

AD is a progressive neurodegenerative disorder distinguished by cognitive decline, memory impairment, and alterations in behavior. It is the leading cause of dementia, affecting around 50 million people worldwide, and this number is expected to triple by 2050 due to an aging population (Lord and Cruchaga, 2014; Xiong et al., 2023). The impact of the disease extends beyond the individuals diagnosed, imposing considerable emotional and psychological burdens on patients and their families, while also presenting significant economic challenge (Johri, 2021). Despite extensive research, the precise etiology of AD remains elusive, and existing therapeutic options are confined to symptomatic treatments that do not arrest disease progression (Sengupta and Kayed, 2022). This highlights the urgent necessity for the development of novel therapeutic strategies aimed at targeting the fundamental mechanisms of the disease.

Astrocytes have recently attracted significant attention for their potential involvement in the pathogenesis of AD. These glial cells are essential for maintaining neuronal health, regulating the integrity of the blood–brain barrier, and modulating synaptic activity (Yang et al., 2022; Patani et al., 2023). In AD, astrocytes become reactive, proliferate, and increase inflammatory mediators, worsening neuronal damage and aiding Aβ plaque formation (Balázs and Kovács, 2021). Research indicates that astrocytes play a significant role in the clearance of amyloid-beta (Aβ) and tau proteins, which are critical markers of AD pathology (Werner et al., 2015). Furthermore, astrocytic dysfunction has been associated with other neurodegenerative disorders, including Parkinson’s disease and amyotrophic lateral sclerosis, underscoring their extensive involvement in neurodegenerative processes (Larner, 2010; Arranz and De Strooper, 2019).

Research on astrocytes in AD has elucidated that these glial cells can assume both neuroprotective and neurotoxic roles contingent upon the specific context. Astrocytes can release neurotrophic factors to support neuron survival or pro-inflammatory cytokines that lead to neuroinflammation and synaptic dysfunction (Patani et al., 2023). The dual functionality of astrocytes in AD implies that targeting their activity may represent a viable therapeutic strategy. Nonetheless, the exact mechanisms through which astrocytes affect the progression of AD remain inadequately elucidated, thereby warranting comprehensive investigation into their multifaceted roles.

In this study, we conducted an extensive bibliometric analysis to investigate the research status pertaining to astrocytes in AD. Utilizing data extracted from the Web of Science Core Collection database, we examined 5,500 pertinent publications covering the period from 2000 to 2024. Our analysis encompassed collaboration networks, keyword co-occurrence, and trends in research themes. To visualize and interpret the data, we employed tools such as VOSviewer, CiteSpace, and the R package “bibliometrix.” These tools facilitated the provision of insights into the evolving research status and the identification of key areas of interest.

The findings from this study are intended to augment our comprehension of the role of astrocytes in AD and to inform future research fields. By delineating research trends, we aspire to contribute to the development of targeted therapeutic interventions aimed at modulating astrocytic activity, with the potential to alter the progression of AD.

## Methods

### Data source and search strategy

**Figure 1:**
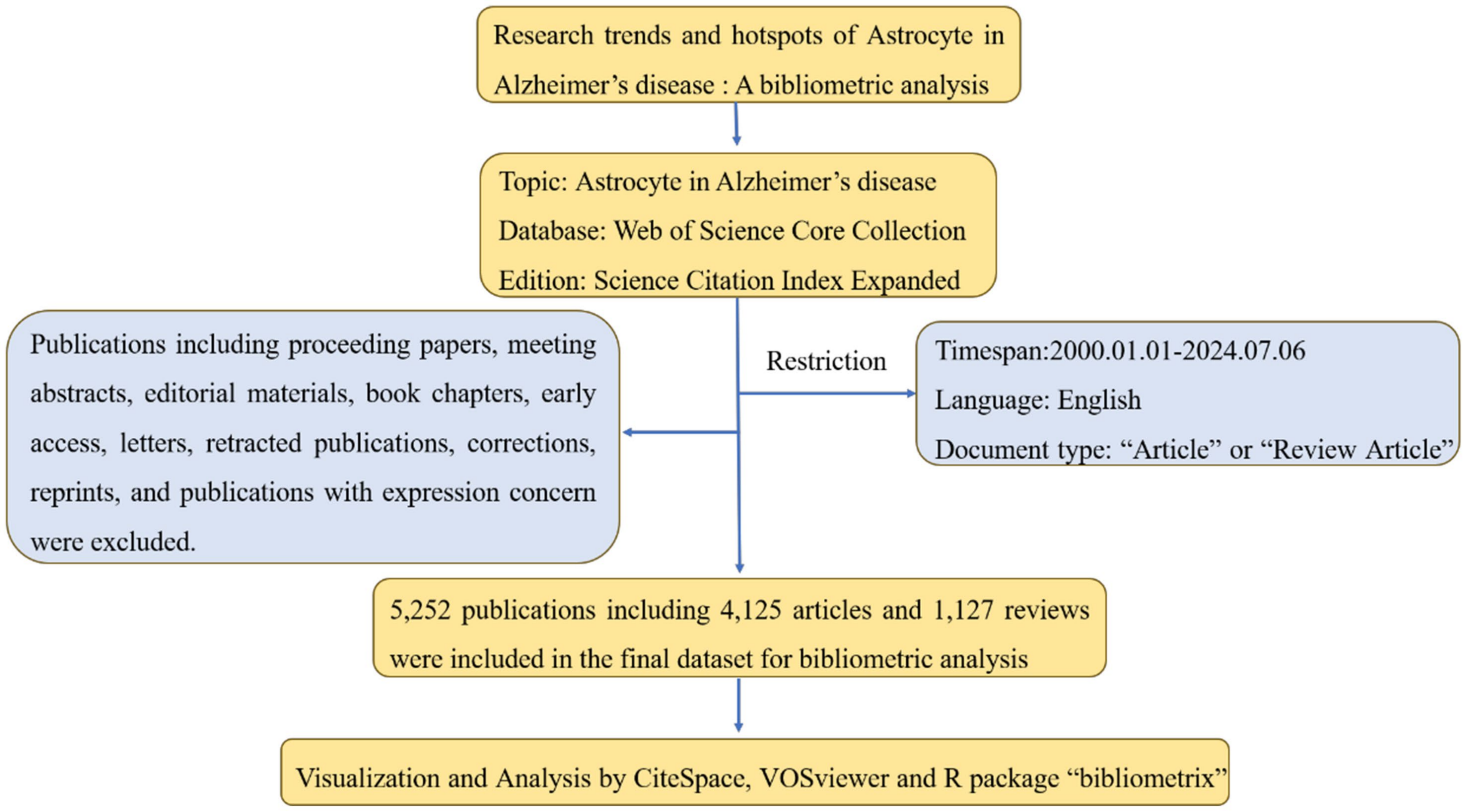
Details of the literature searching and data filtrating.

In this analysis, we used the Web of Science core collection database (WOSCC), which is widely accepted as a high-quality database suitable for bibliometric analysis (Wang et al., 2022). As one of the most extensive and detailed online repositories globally, WOSCC provides an extensive array of authoritative and highly referenced scientific research and evaluations (Gan et al., 2022). The study period was set from 2000 to 2024 (Date of retrieval: 2000.01.01; Date of retrieval deadline: 2024.07.06) and articles must be original. The search query was shown as follows: TS = (“Alzheimer’s disease” OR “Alzheimer” OR “alzheimer’s disease (ad)” OR “alzheimers-disease” OR “Alzheimer Diseases” OR “Alzheimers Diseases”) AND TS = (“Astrocyte” OR “Astrocyte Cell” OR “Astrocyte Cells” OR “Astrocytes”). In addition, only articles and reviews written in English were extracted, as well as restricting the type and language of the documents. A complete literature search and download of data were completed by 6th July 2024, so that we could avoid bias due to frequently updated databases. We exported all articles as TXT files and the data included information on the author, the article title, the journal title, the year the article was published, the institutions, keywords, and citation frequency. Among the thousands of search results, we sorted 5,252 publications: 4,125 articles and 1,127 reviews. Data acquisition and retrieval strategies are shown in Figure 1.

### Bibliometric and visualization analysis

Bibliometric analysis is typically employed to utilize mathematical and statistical techniques to examine research findings, aiming to derive valuable insights and uncover prevailing trends across a broad publication (Yu et al., 2022). Bibliometric insights into countries, institutions, journals, authors, keywords, and references, providing comprehensive data on these aspects (Liao et al., 2023). Furthermore, visualization enables us to assess the current progress of research in a particular field and predict its trends and hotspots (Wang et al., 2021). The three most frequently used tools for bibliometric analysis are VOSviewer, CiteSpace, and Bibliometrix (Dai et al., 2022).

VOSviewer (version 1.6.20) is a bibliometric analysis tool that extracts essential data from numerous publications, and this software is utilized to create collaboration networks, co-citation networks, and cooccurrence networks (Bevilacqua et al., 2024). A number of analyses are performed by the software, including an analysis of countries and institutions, an analysis of journals and co-cited journals, an analysis of authors and co-cited authors, and an analysis of keywords co-occurrences. Additionally, CiteSpace (version 6.3.1) is a widely used bibliometric analysis software that provides insights into research hot spots and evolution processes (Li et al., 2022), it was developed by Professor Chaomei Chen using the Java programming language (Chen, 2004). The mapping of visual knowledge graphs is conducted in accordance with CiteSpace’s core methodology, encompassing stages such as temporal segmentation, filtering by thresholds, constructing models, pruning redundant information, amalgamating related elements, and visual representation. During our research, a CiteSpace analysis was conducted to map the keywords timeline graph and to analyze keywords and references with strong citation bursts. Finally, the R package “bibliometrix” (version 4.2.3) was used to analyze thematic evolution of publications on astrocytes in AD and construct global distribution networks. The initial interpretation of the data is performed through the “biblioAnalysis ()” command and the “summary ()” function in the “bibliometrix” package. Bibliometrix provided the main information on the publications, it includes a comprehensive summary of all published works yearly scientific output, geographical distribution, affiliated organizations, local influence, author demographics, and a word cloud analysis (Zhang et al., 2023; Olaleye et al., 2023).

## Results

### Publication output analysis

**Figure 2:**
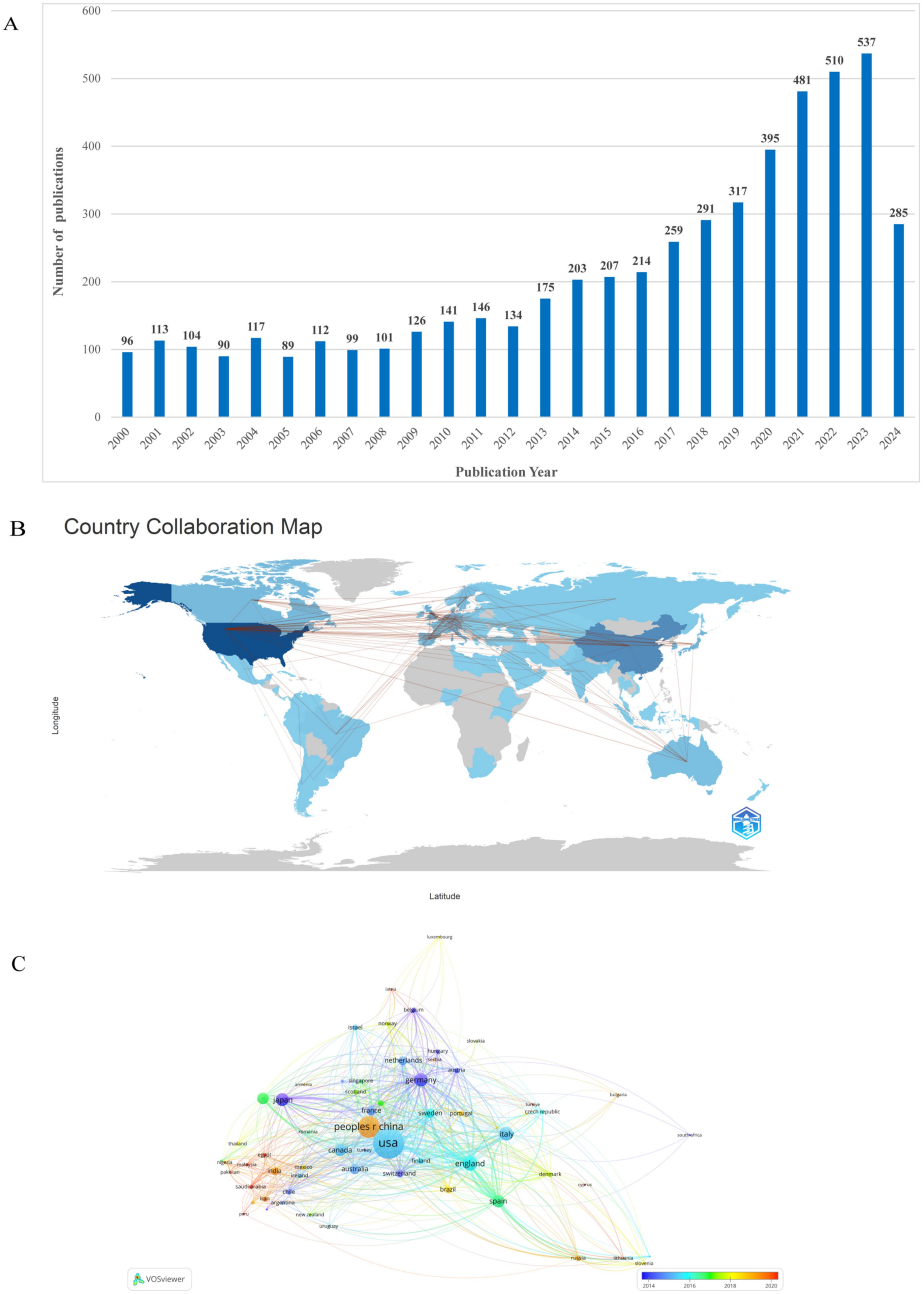
**(A)** The trends of annual publications on the topic of astrocyte in Alzheimer’s disease from 2000 to 2024. **(B)** Publications distributed and collaborated between countries/regions. **(C)** Distributions of countries/regions and collaboration network.

Our search strategy yielded a total of 5,252 publications sourced from the Web of Science core collection online database, covering the period from January 1, 2000, to July 6, 2024. Among these, 4,125 papers (78.45%) are classified as original research articles, while 1,127 papers (21.55%) are categorized as review articles. As illustrated in Figure 2A, there is a consistent annual increase in publication output within this field, indicating a general upward trajectory. Notably, from 2000 to 2016, the literature concerning AD exhibited a steady rise without significant breakthroughs. In contrast, between 2016 and 2023, there was a marked surge in the number of pertinent research articles, escalating from 214 in 2016 to 537 in 2023. This increase underscores a significant advancement in the research on astrocytes in the context of AD, reflecting a notable trend of rapid development.

### Analysis of country and region output

By utilizing bibliometrix and VOSviewer, we are able to visually analyze countries and regions with more than or equal to 5 publications, and build a collaborative network based on how many publications there are in each country and how they are related. All publications on astrocyte research in AD were published by 97 countries or regions. The Figures 2B,C shows the network of national collaborations related to astrocytes in AD. For instance, China engages in significant collaboration with the United States, Japan, Australia, Canada, and Japan, while the United States actively partners with China, England, Canada, and Sweden. The ten countries/regions with the highest contributions are listed in Table 1. Among the countries, the most significant contributor was the USA (*n* = 1,468, 26.7%), followed by China (*n* = 836, 15.2%), Italy (*n* = 288, 5.2%), and United Kingdom (*n* = 263, 4.8%), all of which hold important positions in this area of research. Finally, the combined number of publications from China and the USA accounted for almost half of the total (41.9%), indicates their significant influence in this field.

**Table 1:** Top 10 countries/regions publishing research related to astrocyte in AD.

Rank	Country/region	Publications	Citations	Average article citations
1	USA	1,468 (26.7%)	111,833	76.20
2	China	836 (15.2%)	20,976	25.10
3	Italy	288 (5.2%)	11,311	39.30
4	United Kingdom	263 (4.8%)	17,507	66.60
5	Japan	259 (4.7%)	10,598	40.90
6	Korea	252 (4.6%)	10,769	42.70
7	Germany	218 (4%)	14,146	64.90
8	Canada	186 (3.4%)	9,497	51.10
9	Spain	186 (3.4%)	7,984	42.90
10	Australia	129 (2.3%)	7,442	57.70

### Analysis of institutions

**Figure 3:**
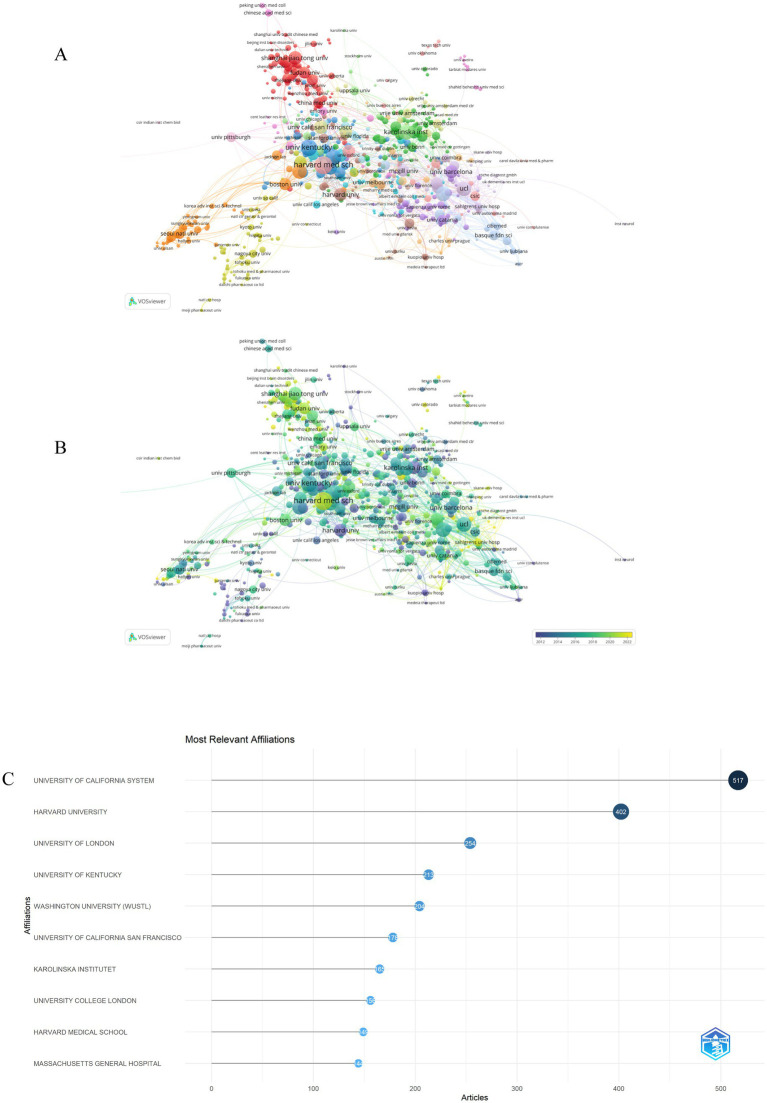
An analysis of collaborations between different institutions. **(A)** Institution co-authorship network map generated by VOSviewer, the node size denotes the cooperative activity, the larger the node, the more cooperation the institutions had with others. **(B)** Institution co-authorship overlay map generated by VOSviewer, the color of each circle indicated the institution’s average publication year, the lighter the color of the node, the more recent its average publication time. **(C)** The top 10 institutions that published most of astrocytes in Alzheimer’s disease-related articles.

Regarding institutional affiliations, a total of 4,462 organizations have contributed to the publication of 5,252 research articles focused on astrocytes in the context of AD. Figure 3C delineates the top 10 institutions based on the volume of published articles. Notably, five institutions have each published over 200 articles. The leading contributors include the University of California system (*n* = 517, 9.84%) and Harvard University (*n* = 402, 7.65%), followed by the University of London (*n* = 254, 4.84%), the University of Kentucky (*n* = 213, 4.06%), and Washington University (*n* = 204, 3.88%). To find out which research institutions were involved in astrocyte research in AD, VOSviewer was used for visualizing the network map. As shown in Figure 3A, a larger circle indicated that the institution had published more articles, and links between institutions indicated collaborative publications. The findings of the study revealed that the University of California system stands out as the foremost institution in terms of research productivity, exhibiting the most robust overall correlation strength within this domain, closely followed by Harvard University. As illustrated in Figure 3B, institutions such as Harvard University, Washington University, the University of California system, the University of Kentucky, and the University of California San Francisco are predominantly represented in lilac and blue hues, signifying their early engagement in this research area. In contrast, Chinese institutions, including Shanghai Jiao Tong University, Fudan University, Peking University, Capital Medical University, and Nanjing Medical University, are primarily depicted in light green and pale yellow shades, reflecting either their relatively recent involvement in the field or the substantial volume of articles they have produced in recent times. The chart further indicates that Harvard Medical School is represented by the largest circle nodes, suggesting that these institutions are emerging as significant players poised to lead future research endeavors and contribute substantially to the advancement of this field.

### Analysis of journals and co-cited journals

**Figure 4:**
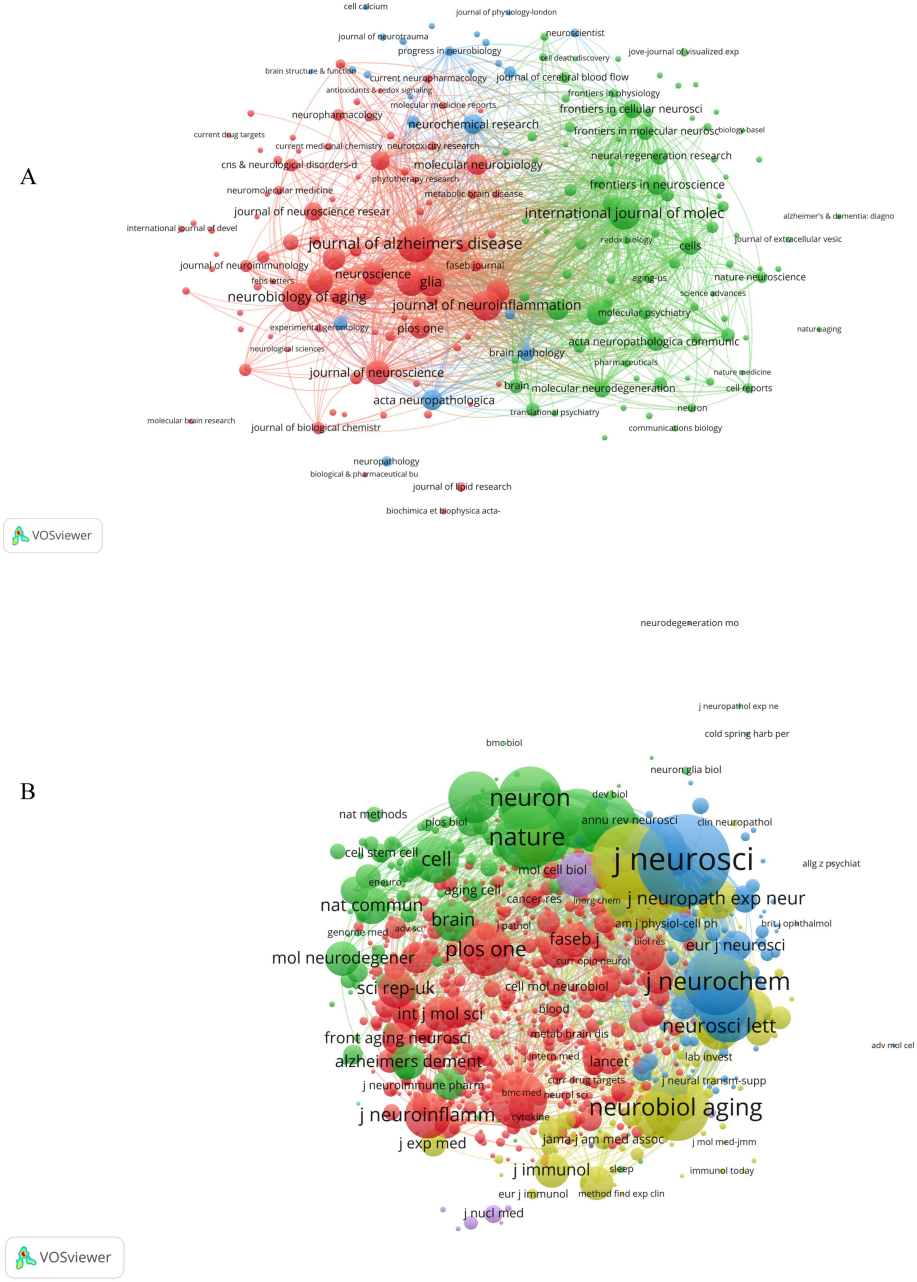
Analysis of journals and co-cited journals. **(A)** Clustering of co-citations among journals. Nodes represent journals, and lines represent co-cited relationships. Node size is proportional to co-cited link strength, and node color indicates cluster membership. **(B)** The bibliographic coupling network of journals related to astrocyte in AD. Circles in the figure represent journals, and each circle’s size indicates how many publications are published in that journal.

The WoSCC search showed that a total of 893 journals participated in the publication of astrocyte in AD. The journal coupling method facilitates the analysis of relationships among journals, aiding in their categorization and revealing the internal knowledge structure of a discipline. This approach is particularly beneficial for researchers seeking to efficiently select suitable journals for submission. Clusters are formed based on citation frequencies and mutual citation patterns, which provide insights into the knowledge structure and collaborative networks within specific research domains. And our collaborative networks were constructed in each journal based on the number of publications greater than or equal to 5. As shown in Figure 4A, 197 of them met the thresholds and the sources analysis was visualized. Figure 4B shows the journal coupling analysis was conducted on 893 journals that were put into three clusters, and the representative journals were: the “Journal of Alzheimer’s Disease” (Cluster 1 in red) is dedicated to a wide array of research topics concerning AD and the journal covers research on the etiology, pathogenesis, diagnosis, treatment, and related neurobiology and molecular biology of AD. The International Journal of Molecular Sciences (cluster 2 is shown in green) covers all aspects of molecular biology, including basic and applied research. In the field of AD, the journal may focus on a variety of research areas of astrocytes in the disease, such as the role of astrocytes in Aβ protein metabolism, inflammatory responses, regulation of synaptic function, and interactions with microglia. And Neurochemical research (cluster 3 is shown in blue) may focus on include cell activation status, interaction with amyloid, metabolic function, signaling, therapeutic potential, and role in the early stages of the disease in the research areas of astrocytes in AD.

In addition, we listed the 10 most prolific journals, as shown in Table 2, AD was the most prolific journal, with 215 articles (4.09%), followed by the International Journal of Molecular Sciences with 163 articles (3.10%), and the Journal of Neuroinflammation with 142 articles (2.70%). Among the ten journals that have published the highest number of papers, four are categorized within the Q1, five fall into Q2, and one is classified as Q3. Notably, Neuroinflammation has the highest impact factor (IF) of 9.3, placing it within the Q1 category. In terms of citation frequency, six of the ten most cited journals are also classified in Q1, while four are in Q2, underscoring their esteemed status and influence within their respective disciplines. The impact of a journal is predominantly assessed by the volume of citations it garners, as these citations serve as a metric for how frequently its articles are referenced and utilized by scholars and researchers. Within the top ten cited journals, the Journal of Neuroscience leads with an impressive total of 16,738 citations, followed by the Proceedings of the National Academy of Sciences of the United States of America with 11,637 citations, and the Journal of Biological Chemistry with 11,297 citations. This data highlights the substantial influence these journals exert on the field of AD research.

**Table 2:** Top 10 journals and co-cited journals related to astrocytes in Alzheimer’s.

Rank	Journal	Publications	IF (2023)	JCR quantile	Rank	Cited journal	Citations	IF (2023)	JCR quantile
1	Journal of Alzheimers Disease	215	3.4	Q2	1	J Neurosci	16,738	4.4	Q1
2	International Journal of Molecular Sciences	163	4.9	Q2	2	P Natl Acad Sci USA	11,637	9.4	Q1
3	Journal of Neuroinflammation	142	9.3	Q1	3	J Biol Chem	11,297	4	Q2
4	Neurobiology of Aging	136	3.7	Q2	4	J Neurochem	9,057	4.2	Q2
5	Journal of Neurochemistry	122	4.2	Q2	5	Nature	8,420	11.28	Q1
6	Glia	120	5.1	Q1	6	Neurobiol Aging	8,348	3.7	Q2
7	Brain Research	100	2.7	Q3	7	Neuron	7,879	14.7	Q1
8	Frontiers in Aging Neuroscience	99	4.1	Q2	8	Glia	7,271	5.1	Q1
9	Journal of Neuroscience	93	4.1	Q1	9	Science	6,955	44.7	Q1
10	Neurobiology of Disease	86	5.1	Q1	10	J Alzheimers Dis	6,402	3.4	Q2

Moreover, the network visualization for the journal co-citation network was created using VOSviewer, and the larger node indicates that the journals are more influential. Figure 4B presents the network visualization of the journal co-citation network, generated by VOSviewer with a citation threshold of 20, forming a total of 1,348 nodes with 399,432 links in the graph. In this visualization, journals that are frequently co-cited are highlighted with larger font sizes, making them more conspicuous within the network depicted in Figure 4B. The top three journals with the largest TSL values were Journal of Neuroscience (TSL = 2,007,063), Proceedings of the National Academy of Sciences of the United States of America (TSL = 1,343,934), and Journal of Biological Chemistry (TSL = 1,217,336), these three journals had the strongest correlation with other journals in the field.

### Analysis of authors and co-cited authors

**Figure 5:**
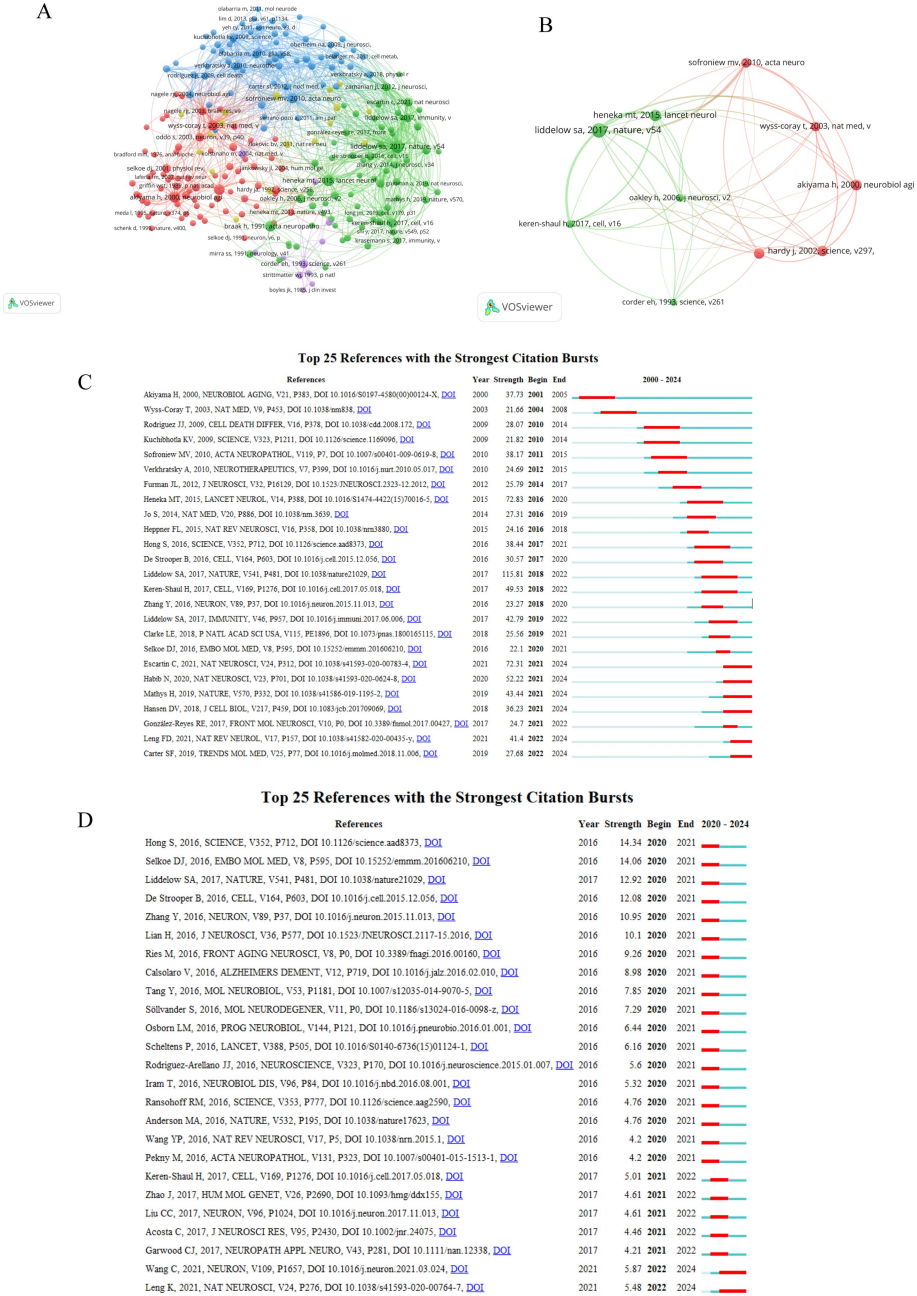
Analyzing literature. **(A)** The VOSviewer-generated co-citation visualization map among references pertinent to astrocyte research in AD. After clustering these references, 5 major clusters were formed. Different colored nodes represent different clusters. Cluster 1: (in red) included 73 references, Cluster 2 (in green) included 68 references, Cluster 3 (in blue) included 64 references, Cluster 4 (in yellow) included 17 references, Cluster 5 (in purple) included 11 references. **(B)** The top ten co-cited references. **(C)** The top 25 references with the greatest citation bursts during the period from 2000 to 2024. **(D)** The top 25 references with the strongest citation bursts during the period from 2020 to 2024.

**Figure 6:**
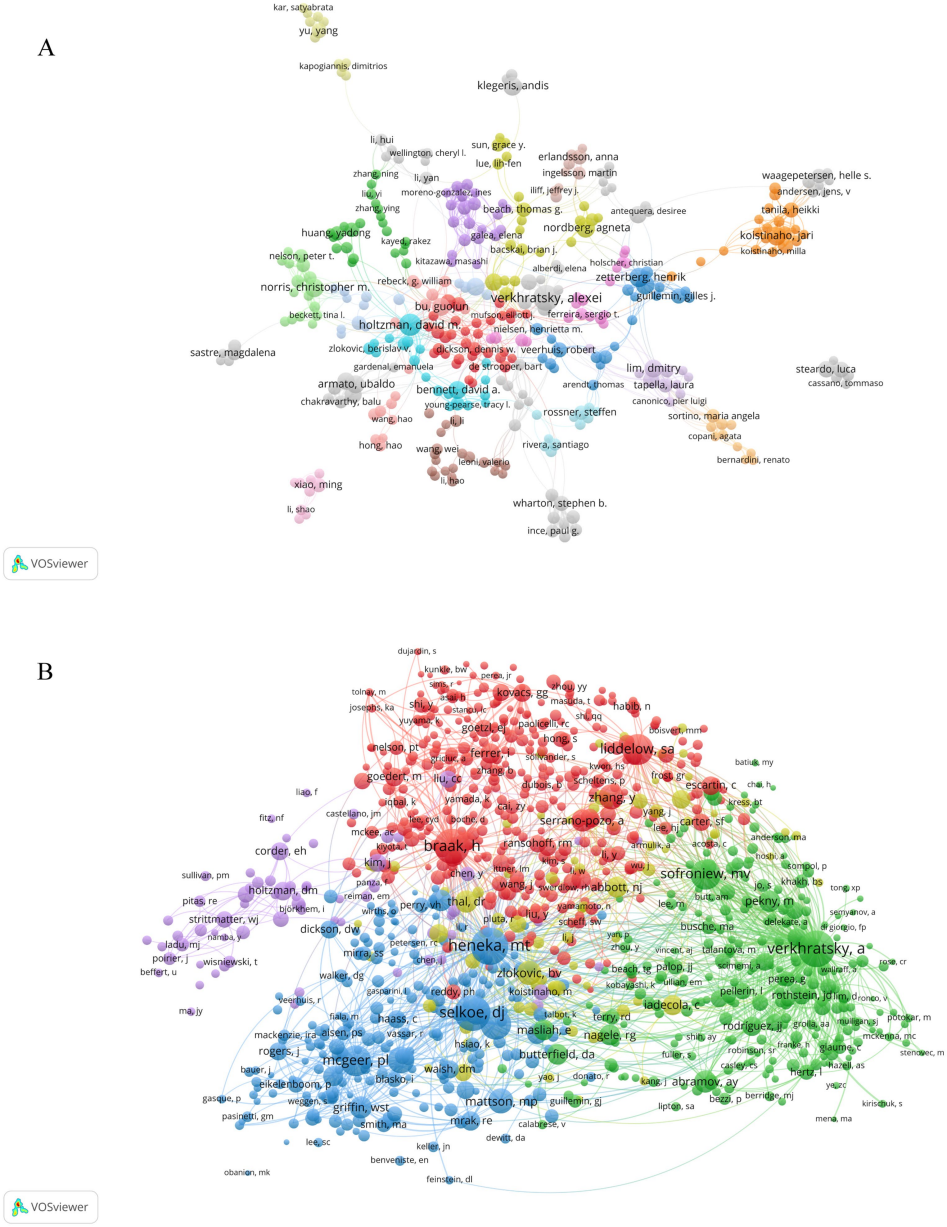
Analysis of active authors using network visualization. **(A)** Analysis of author collaboration in a network visualization map. **(B)** Analysis of co-cited authors in a network visualization map. Each node represents an author or co-cited author, with node size indicating their citations or documents, and the lines between authors representing their collaborations.

A total of 26,375 authors participated in research of astrocyte in AD. Figure 5A shows the collaborative network that we build based on authors who have published five or more papers. As shown in Table 3, the top 10 authors are identified based on the number of publications and citations they have received, along with their contributions to advancing our understanding of AD. Among the authors, the three most prolific publishers are VERKHRATSKY A, with 51 publications, followed by HOLTZMAN DM with 36, and FERRER I and ZHANG Y, each with 32 papers. Among the top 10 co-citation authors, VERKHRATSKY A (1,585 citations) ranks first, followed by RODRÍGUEZ JJ (834 citations) and HOLTZMAN DM (815 citations). The VOSviewer was used to visualize a collaborative network of co-authorship and citation networks between authors, the co-authors formed 28 clusters (Figure 6A). Each node stands for a researcher, with the size of the circle indicating the number of papers they have published. The lines that connect the circles denote the collaborative relationships among researchers, and these connections are colored differently to denote various clusters. As illustrated in Figure 6B, there exists a notable co-occurrence relationship among the authors and their co-cited counterparts. Authors who demonstrate high productivity are observed to frequently co-occur with others. The analysis reveals that the co-cited authors can be categorized into five distinct clusters. Among them, VERKHRATSKY A stands out with the highest number of co-citations, followed closely by RODRÍGUEZ JJ and HOLTZMAN DM. These individuals constitute the central research contributors within the discipline and are recognized for their esteemed academic standing.

**Table 3:** Top 10 authors and co-cited authors related to astrocyte in AD.

Rank	Author	Counts	Percentage (%)	Rank	Co-cited author	Citations
1	Verkhratsky A	51	0.9	1	Verkhratsky A	1,585
2	Holtzman Dm	35	0.6	2	Rodríguez Jj	834
3	Ferrer I	32	0.6	3	Holtzman Dm	815
4	Zhang Y	32	0.6	4	Veerhuis R	722
5	Li Y	30	0.5	5	Liddelow Sa	648
6	Liu Y	29	0.5	6	Dawson Tm	605
7	Zetterberg H	27	0.5	7	Dawson Vl	605
8	Zhang L	27	0.5	8	Barres B	584
9	Blennow K	26	0.5	9	Kumar M	584
10	Hong Jt	26	0.5	10	Panicker N	584

### Analysis of references and co-cited references

It is critical for researchers to understand which works have gained the most attention and citations, so they can build upon existing knowledge and concepts. Table 4 displays top 10 most frequently cited articles related to astrocyte in AD. “Neurotoxic reactive astrocytes are induced by activated microglia” is the most cited article (4,378 citations). This study showing that activated microglia can induce reactive astrocytes with neurotoxic properties is of significant scientific significance, offering insight into neurodegenerative disease pathogenesis (Liddelow et al., 2017). Second most cited article is “Inflammation and Alzheimer’s disease” with 3,566 citations. The objective of this study is to enhance the comprehension of the immune-modulatory and inflammatory processes associated with AD, while also contributing to the formulation of innovative anti-inflammatory strategies intended to decelerate the progression of the disease (Akiyama et al., 2000). The third most-frequently cited article was “A paravascular pathway facilitates CSF flow through the brain parenchyma and the clearance of interstitial solutes, including amyloid *β* (Iliff et al., 2012),” with 2,985 citations. The study identified a mechanism that flow of CSF through the parenchyma of the brain is facilitated by paravascular pathways, which clear interstitial solutes including amyloid, and it offers a number of therapeutic possibilities for improving solute clearance and treating brain diseases, and it highlights the importance of the accessory vascular pathway in the AD (Iliff et al., 2012). It is widely recognized that a citation relationship typically involves two or more references being cited simultaneously by one or more papers. By analyzing these citation patterns, researchers can identify influential papers, trace the evolution of ideas, and uncover the interconnectedness of various studies. This approach can provide valuable insights into the structure and dynamics of academic knowledge (Wu et al., 2021). Over the past two decades, 205,521 co-cited references have been published on astrocyte research in AD. All of the top 10 co-cited references (Table 5) were cited at least 200 times, and one reference was cited more than 500 times. In order to construct a map of co-citations, references with a co-citation count of at least 60 were selected, a total of 203 references were selected for analysis of co-citations (Figure 5A). There were different colors for different clusters of references. The first cluster (in red) included 73 references, while the second cluster (in green) included 68 references. Cluster 3 (in blue) included 64 references, Cluster 4 (in yellow) included 17 references and Cluster 5 (in purple) included 11 references. As shown in Figure 5B, the top ten co-cited references are mostly related to pathology, genetics, molecular biology, and cell biology. CiteSpace’s analytical capabilities were used to identify citation bursts in the field of “astrocyte-Alzheimer’s disease,” highlighting studies that have garnered significant scholarly attention. Figure 5C illustrates an examination of the 25 most cited references, highlighting notable surges in citations that underscore their substantial influence. By scrutinizing the articles exhibiting the highest levels of explosive citation intensity, we can gain insights into the trajectory of research advancement. Among these, the publication titled “Neurotoxic reactive astrocytes are induced by activated microglia,” authored by Shane A. Liddelow and colleagues in 2017 in the journal Nature, exhibited the most pronounced citation burst (strength = 115.81), with the surge occurring between 2019 and 2022 (Liddelow et al., 2017). This was closely followed in impact by the works of Heneka et al. (2015) and Akiyama et al. (2000). It can be helpful to identify key studies that have received significant scholarly attention and determine when these bursts occurred. The analysis of co-citation references and identification of works that are frequently cited together allows researchers to identify potential collaborators who have similar research interests, improving their ability to collaborate.

**Table 4:** Top 10 articles with the most citations.

Rank	Literature	Cited frequency	Author	Year	Journal	IF (2023)
1	Neurotoxic reactive astrocytes are induced by activated microglia	4,378	Shane A Liddelow	2017	Nature	50.5
2	Inflammation and Alzheimer’s disease	3,655	H Akiyama	2000	Neurobiol Aging	3.7
3	A paravascular pathway facilitates CSF flow through the brain parenchyma and the clearance of interstitial solutes, including amyloid β	2,569	Jeffrey J Iliff	2012	Science Translational Medicine	15.8
4	The blood–brain barrier in health and chronic neurodegenerative disorders	2,407	Berislav V Zlokovic	2008	Neuron	14.7
5	Neurovascular regulation in the normal brain and in Alzheimer’s disease	1,689	Costantino Iadecola	2004	Nature Reviews Neuroscience	28.7
6	The Cellular Phase of Alzheimer’s Disease	1,143	Bart De Strooper	2016	Cell	45.5
7	Microglia in Alzheimer’s disease	1,063	David V Hansen	2018	Journal of Cell Biology	7.4
8	Primary age-related tauopathy (PART): a common pathology associated with human aging	963	John F Crary	2014	Acta Neuropathologica	9.3
9	Apolipoprotein E controls cerebrovascular integrity via cyclophilin A	946	Robert D Bell	2012	Nature	50.5
10	Neuroinflammation in neurodegenerative disorders: the roles of microglia and astrocytes	933	Hyuk Sung Kwon	2020	Transl Neurodegener	10.8

**Table 5:** The top 10 articles with the most co-citations.

Rank	Co-cited literature	Co-cited counts	Author	Year	Journal	IF (2023)
1	Inflammation and Alzheimer’s disease	348	H Akiyama	2000	Neurobiology of Aging	3.7
2	Neuropathological stageing of Alzheimer-related changes	328	H Braak	1991	Acta Neuropathologica	9.3
3	Gene dose of apolipoprotein E type 4 allele and the risk of Alzheimer’s disease in late onset families	199	E H Corder	1993	Science	44.7
4	The amyloid hypothesis of Alzheimer’s disease: progress and problems on the road to therapeutics	329	John Hardy	2002	Science	44.7
5	Neuroinflammation in Alzheimer’s disease	369	Michael T Heneka	2015	Lancet Neurology	46.5
6	A Unique Microglia Type Associated with Restricting Development of Alzheimer’s Disease	221	Hadas Keren-Shaul	2017	Cell	45.5
7	Neurotoxic reactive astrocytes are induced by activated microglia	514	Shane A Liddelow	2017	Nature	50.5
8	Intraneuronal beta-amyloid aggregates, neurodegeneration, and neuron loss in transgenic mice with five familial Alzheimer’s disease mutations: potential factors in amyloid plaque formation	224	Holly Oakley	2006	Journal of Neuroscience	4.4
9	Astrocytes: biology and pathology	306	Michael V Sofroniew	2010	Acta Neuropathologica	9.3
10	Adult mouse astrocytes degrade amyloid-beta *in vitro* and *in situ*	289	Tony Wyss-Coray	2003	Nature Medicine	58.7

Furthermore, we utilized CiteSpace to identify the top 25 references with the strongest citation bursts during the period from 2020 to 2024, with the goal of identifying and monitoring the latest research trends and focal points concerning astrocytes in AD. Figure 5D illustrated that, since 2020, the strongest citation burst originated from the paper by Hong et al. on Science in 2016, exhibited the most pronounced citation burst (strength = 14.34), this study suggests that modulating astrocyte activation or the factors they release could potentially improve symptoms and slow the progression of AD (Hong et al., 2016). Followed by the article by Dennis J Selkoe et al. on Embo Molecular Medicine in 2016 (strength = 14.06), the literature emphasizes the important role of astrocytes in AD, particularly in terms of their potential to clear Aβ and restore cognitive functions, and the study suggest that by modulating the autophagy process of astrocytes, it is possible to reduce Aβ oligomers in the brain and improve memory and cognitive functions, offering new strategies and potential therapeutic targets for the treatment of AD (Selkoe and Hardy, 2016). The third most prominent is the article by Shane A. Liddelow et al., published in Nature in 2019, with a strength of 12.92 (Liddelow et al., 2017).

### Analysis of keywords

**Figure 7:**
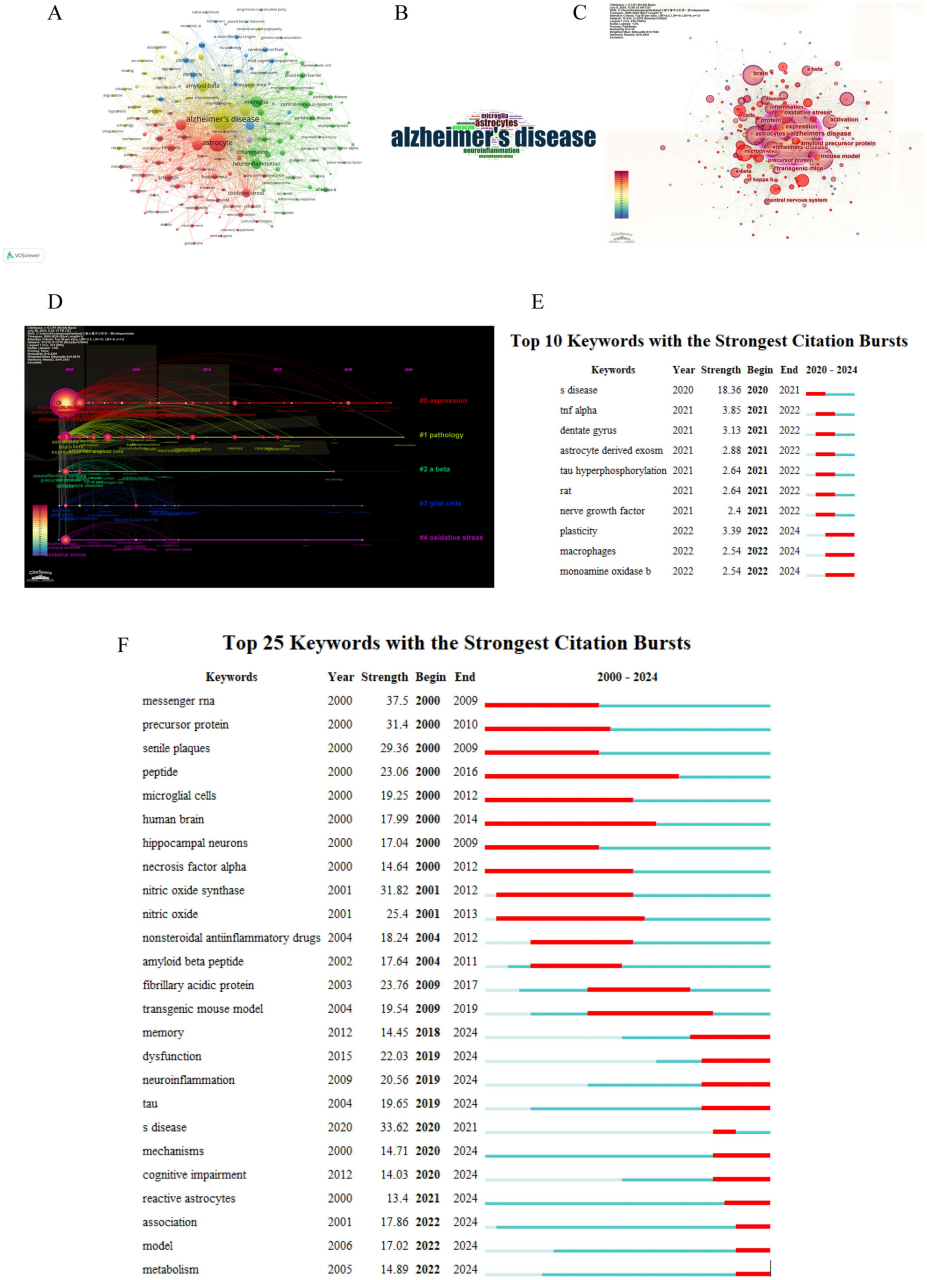
Keywords cluster analysis of correlation between metabolomics AD and astrocyte. **(A)** Keyword cluster analysis. Cluster 1 (in red) included 62 keywords. Cluster 2 (in green) included 62 keywords. Cluster 3 (in blue) included 40 keywords. Cluster 4 (in yellow) included 33 keywords. Cluster 5 (in purple) included 1 keyword. **(B)** The keyword cloud of the retrieved articles. **(C)** Co-occurrence network of keywords. **(D)** Clustering timeline of keywords. **(E)** Keywords burst analysis indicated by the map of “Top 10 Keywords with the Strongest Citation Bursts” during the period from 2020 to 2024. **(F)** Keywords burst analysis indicated by the map of “Top 25 Keywords with the Strongest Citation Bursts” during the period from 2000 to 2024.

Identifying key terms within the article provides us with insight into the topic of the article (van Eck and Waltman, 2017). By identifying these keywords and their clusters, we can identify current research hotspots and frontiers, and suggest future directions (Dotsika and Watkins, 2017). We used VOSviewer software to perform co-occurrence clustering analysis, with a minimum keyword occurrence threshold of 50 instances. Keywords meeting these criteria were included in the graph, and each circle’s size was positively correlated with keyword frequency. The visualized network was created using 184 keywords (after removing duplicates) from the initial pool of 16,027 keywords (Figure 7A). As shown in Figure 7A, the key-word clustering analysis revealed 5 different clusters, the first cluster (in red) included 62 keywords, with “astrocyte,” “oxidative stress” and “brain” dominating the red cluster. While the second cluster (in green) included 62 keywords, it contains primary keywords such as “microglia,” “inflammation,” and “neuroinflammation.” Cluster 3 (in blue) included 40 keywords, Cluster 4 (in yellow) included 33 keywords and Cluster 5 (in purple) included 1 keyword. The keywords “tau,” “neurodegeneration” and “amyloid-beta” are also important. In order to visualize keyword occurrence and frequency.

Bibliometrix was applied to the article’s keywords, with a minimum keyword occurrence threshold of 10 instances. In Figure 7B, the font size corresponds to the frequency of keywords in retrieved articles. First of all, keywords ranked in the top three were “alzheimer’s disease,” “astrocyte,” and “amyloid-beta.” Then, microglia,” “brain,” “mutations,” “expression,” and “neuroinflammation” followed closely behind. Analysis of co-occurrences reveals themes and concepts that relate to keywords.

As shown in Figure 7C, a co-occurrence analysis chart reflects frequency of keywords, utilizing CiteSpace to analyze the co-occurrence of “astrocyte- Alzheimer’s disease” from 1 January 2000, to 6 July 2024. Sizes of spheres indicate the cumulative size of spheres on annual rings, proportional to keyword usage frequency. Earlier keyword appearances are purple, later keyword appearances are red, and overlapping colors indicate citations in the corresponding years are overlapping. In addition, the lines connecting the spheres represent connections among literature citing each other. Figure 7C shows that the most frequently co-occurring keyword is “alzheimer’s disease,” followed by “astrocyte.”

CiteSpace utilized the timeline as the analytical node to create a keyword time chart that illustrates the chronological progression of keywords. This timeline view provides a more lucid representation of historical study findings, patterns, and intrinsic connections in each group. Every node signifies a unique keyword, where the size of the node is directly proportional to its frequency of appearance. We utilized CiteSpace to acquire 5 clusters and created a clustering timeline, each represented by a horizontal line (Figure 7D). The map of clustering time can additionally display the manifestation of every cluster and identical colors denoted identical clusters. We selected tree ring type nodes; the color stripes around each node symbolized the frequency of this keyword in each respective year. For example, the “pathology” node was mainly made up of yellow stripes, signifying that this term was often used between the years 2009 and 2015. The color of connecting lines between nodes was set to reflect the average year of co-occurrence. Figure 7D depicts the chronological perspective of grouped keywords, offering a thorough visualization of the evolution and trends of research clusters. Examining the time development of keywords, in 2000, high-frequency keywords such as “Alzheimer’s disease,” “astrocyte,” “amyloid-beta,” and “oxidative stress” signified the commencement of research at an early stage with significant results.

The CiteSpace algorithm identifies surges in research subjects. Figure 7F shows the top 25 most cited keywords. Among the keywords with the highest burst intensity, “messenger RNA” (37.5) ranked highest, followed by “Alzheimer’s disease” (33.62) and “nitric oxide synthase” (31.82). The keyword with the longest duration was “peptide,” duration ranged from 2000 to 2016, followed by “human brain,” “microglial cells,” “necrosis factor alpha.” Additionally, we utilized CiteSpace to identify the top 10 keywords with the strongest citation bursts during the period from 2020 to 2024 (Figure 7E). The keywords appeared recently with strong citation bursts were “Alzheimer’s disease,” “tnf alpha” and “dentate gyrus,” representing the current research hotspots in this field.

## Discussion

AD represents a significant global health challenge characterized by a progressive deterioration of cognitive functions, memory impairment, and, ultimately, the loss of the ability to perform daily activities independently (Jose et al., 2019). Projections indicate that by 2050, over 15 million individuals worldwide are expected to be affected by this neurodegenerative disorder, thereby imposing considerable emotional and financial burdens on patients, their families, and healthcare systems (Debapriya et al., 2019). Current treatments mainly involve drugs and other methods but are not very effective in slowing AD or improving patient quality of life (Paulo et al., 2022). Therefore, new research is urgently needed to investigate the disease’s underlying mechanisms, especially the role of glial cells like astrocytes.

This study uses bibliometric analysis to examine astrocytes’ role in Alzheimer’s disease, highlighting key trends and research developments from 2000 to 2024 using Web of Science data. Analyzing 5,252 relevant publications, including numerous original studies and reviews, this research underscores the growing scholarly focus on astrocytes in AD pathology (Spotorno et al., 2022). The findings illuminate the growing acknowledgment of the roles of astrocytes in neuroinflammation, metabolism, and neuroprotection in the context of AD (Zulfiqar et al., 2019). This underscores the potential for developing astrocyte-targeted therapeutic strategies to enhance the management of AD.

The bibliometric analysis reveals a significant increase in research dedicated to astrocytes and their roles in AD, with the number of publications rising from 214 in 2016 to 537 in 2023. This upward trend underscores an expanding interest in the topic, corresponding with the increasing global prevalence of AD. It indicates a heightened awareness among researchers regarding the critical role astrocytes play in neuroinflammatory processes and neurodegeneration associated with the disease (Matthew and Jason, 2017; Hamilton et al., 2024). The rise in the number of both original research and review articles suggests a maturation of the field. Furthermore, the expansion in research output can be ascribed to advancements in novel methodologies and models, which facilitate a more profound exploration of astrocyte biology (Krawczyk et al., 2022). This progress holds the potential to develop innovative therapeutic strategies aimed at enhancing the quality of life for patients suffering from AD.

Authorship patterns reveal that a few researchers dominate the field, often collaborating on numerous publications. While such close collaboration may facilitate the establishment of a cohesive vision for future research trajectories, it simultaneously raises concerns about the diversity of perspectives and the potential implications for innovation. To address these challenges, it is crucial to foster the engagement of emerging researchers and to advocate for interdisciplinary collaborations. These initiatives may prevent stagnation in research productivity and stimulate innovative approaches to elucidating the roles of astrocytes in AD.

This study is limited by its exclusive use of the Web of Science, potentially missing key works from databases like PubMed and CNKI, and its focus on English-language publications, which may overlook important research in other languages. The lack of standardized quality assessment criteria could also bias our interpretations. Future research should include diverse databases and languages and apply rigorous quality assessment protocols for a more comprehensive understanding of the field.

## Conclusion

This research offers a comprehensive overview of evolving studies on astrocytes and AD, highlighting publication trends, geographic and institutional contributions, and key authors. These insights will guide future research on Alzheimer’s mechanisms. This analysis provides insights that will guide future research into Alzheimer’s mechanisms. As the field evolves, fostering interdisciplinary collaboration and integrating empirical findings will be essential for turning these insights into meaningful therapeutic advancements for patients.

## Data Availability

The original contributions presented in the study are included in the article/Supplementary material, further inquiries can be directed to the corresponding authors.

## References

[ref1] Akiyama H. Barger S. Barnum S. Bradt B. Bauer J. Cole G. M.. (2000). Inflammation and Alzheimer's disease. Neurobiol. Aging 21, 383–421. doi: 10.1016/S0197-4580(00)00124-X, PMID: 10858586 PMC3887148

[ref2] Arranz A. M. De Strooper B. (2019). The role of astroglia in Alzheimer's disease: pathophysiology and clinical implications. Lancet Neurol. 18, 406–414. doi: 10.1016/S1474-4422(18)30490-3, PMID: 30795987

[ref3] Balázs N. Kovács T. (2021). Heterogeneity of Alzheimer’s disease. Orv. Hetil. 162, 970–977. doi: 10.1556/650.2021.32130, PMID: 34148025

[ref4] Bevilacqua A. Campaniello D. Speranza B. Racioppo A. Sinigaglia M. Corbo M. R. (2024). An update on prebiotics and on their health effects. Food Secur. 13:446. doi: 10.3390/foods13030446, PMID: 38338581 PMC10855651

[ref27] Chen C. (2004). Searching for intellectual turning points: progressive knowledge domain visualization. Proc Natl Acad Sci USA. 101:5303. doi: 10.1073/pnas.0307513100 14724295 PMC387312

[ref5] Dai Z. Xu S. Wu X. Hu R. Li H. He H.. (2022). Knowledge mapping of multicriteria decision analysis in healthcare: a bibliometric analysis. Front. Public Health 10:895552. doi: 10.3389/fpubh.2022.895552, PMID: 35757629 PMC9218106

[ref7] Debapriya G. Nidhi A. Anjali S. Sahil S. (2019). Mitochondrial metabolism: a common link between neuroinflammation and neurodegeneration. Behav. Pharmacol. 30, 641–651. doi: 10.1097/FBP.0000000000000505 31625975

[ref8] Dotsika F. Watkins A. (2017). Identifying potentially disruptive trends by means of keyword network analysis. Technol. Forecast. Soc. Chang. 119, 114–127. doi: 10.1016/j.techfore.2017.03.020

[ref9] Gan P. Pan X. Huang S. Xia H. Zhou X. Tang X. (2022). Current status of coronavirus disease 2019 vaccine research based on bibliometric analysis. Hum. Vaccin. Immunother. 18:2119766. doi: 10.1080/21645515.2022.2119766, PMID: 36494998 PMC9746459

[ref10] Hamilton H. L. Kinscherf N. A. Balmer G. Bresque M. Salamat S. M. Vargas M. R.. (2024). FABP7 drives an inflammatory response in human astrocytes and is upregulated in Alzheimer's disease. Geroscience 46, 1607–1625. doi: 10.1007/s11357-023-00916-0, PMID: 37688656 PMC10828232

[ref11] Heneka M. T. Carson M. J. El Khoury J. Landreth G. E. Brosseron F. Feinstein D. L.. (2015). Neuroinflammation in Alzheimer's disease. Lancet Neurol. 14, 388–405. doi: 10.1016/S1474-4422(15)70016-5 25792098 PMC5909703

[ref12] Hong S. Beja-Glasser V. F. Nfonoyim B. M. Frouin A. Li S. Ramakrishnan S.. (2016). Complement and microglia mediate early synapse loss in Alzheimer mouse models. Science 352, 712–716. doi: 10.1126/science.aad8373, PMID: 27033548 PMC5094372

[ref13] Iliff J. J. Wang M. Liao Y. Plogg B. A. Peng W. Gundersen G. A.. (2012). A paravascular pathway facilitates CSF flow through the brain parenchyma and the clearance of interstitial solutes, including amyloid β. Sci. Transl. Med. 4:147ra111. doi: 10.1126/scitranslmed.3003748 PMC3551275 22896675

[ref32] Johri A. (2021). Disentangling Mitochondria in Alzheimer’s Disease. Int J Mol Sci. 22:11520. doi: 10.3390/ijms222111520, PMID: 34768950 PMC8583788

[ref14] Jose S. L. A. Hector G. M. Gabriel L. C. (2019). Alzheimer’s disease. Handb. Clin. Neurol. 167, 231–255. doi: 10.1016/B978-0-12-804766-8.00013-3 31753135

[ref15] Krawczyk M. C. Haney J. R. Pan L. Caneda C. Khankan R. R. Reyes S. D.. (2022). Human astrocytes exhibit tumor microenvironment-, age-, and sex-related transcriptomic signatures. Neuroscience 42, 1587–1603. doi: 10.1523/JNEUROSCI.0407-21.2021, PMID: 34987109 PMC8883850

[ref16] Larner A. J. (2010). Cholinesterase inhibitors: beyond Alzheimer’s disease. Expert. Rev. Neurother. 10, 1699–1705. doi: 10.1586/ern.10.105 21046692

[ref17] Li M. Wang Y. Xue H. Wu L. Wang Y. Wang C.. (2022). Scientometric analysis and scientific trends on microplastics research. Chemosphere 304:135337. doi: 10.1016/j.chemosphere.2022.135337, PMID: 35714953

[ref18] Liao Y. Wang L. Liu F. Zhou Y. Lin X. Zhao Z.. (2023). Emerging trends and hotspots in metabolic dysfunction-associated fatty liver disease (MAFLD) research from 2012 to 2021: a bibliometric analysis. Front. Endocrinol. 14:1078149. doi: 10.3389/fendo.2023.1078149, PMID: 36761200 PMC9904363

[ref19] Liddelow S. A. Guttenplan K. A. Clarke L. E. Bennett F. C. Bohlen C. J. Schirmer L.. (2017). Neurotoxic reactive astrocytes are induced by activated microglia. Nature 541, 481–487. doi: 10.1038/nature21029, PMID: 28099414 PMC5404890

[ref20] Lord J. Cruchaga C. (2014). The epigenetic landscape of Alzheimer's disease. Nat. Neurosci. 17, 1138–1140. doi: 10.1038/nn.3792, PMID: 25157507 PMC5472058

[ref21] Matthew. N. Jason R. R. (2017). Epigenetic regulation of astrocyte function in neuroinflammation and neurodegeneration. Biochim. Biophys. Acta Mol. basis Dis. 1864, 432–443. doi: 10.1016/j.bbadis.2017.11.004 29113750 PMC5743548

[ref22] Olaleye S. A. Sanusi I. T. Dada O. A. Agbo F. J. (2023). A bibliometric review of global visibility, impact and adoption of electronic invoicing: the past and the future. Heliyon 9:e13726. doi: 10.1016/j.heliyon.2023.e13726, PMID: 36915563 PMC10006452

[ref6] Patani R. Hardingham G. E. Liddelow S. A.. (2023). Functional roles of reactive astrocytes in neuroinflammation and neurodegeneration. Nat Rev Neurol. 19, 395–409. doi: 10.1038/s41582-023-00822-1, PMID: 37308616

[ref23] Paulo C. Valeska M. Jerson L. Della C. M. V. Florindo S. Francisco C. E.. (2022). Treatment of dementia: recommendations of the scientific Department of Cognitive Neurology and Aging of the Brazilian academy of neurology. Dement Neuropsychol. 16, 88–100. doi: 10.1590/1980-5764-DN-2022-S106PT 36533154 PMC9745994

[ref24] Selkoe D. J. Hardy J. (2016). The amyloid hypothesis of Alzheimer's disease at 25 years. EMBO Mol. Med. 8, 595–608. doi: 10.15252/emmm.201606210, PMID: 27025652 PMC4888851

[ref25] Sengupta U. Kayed R. (2022). Amyloid β, tau, and α-Synuclein aggregates in the pathogenesis, prognosis, and therapeutics for neurodegenerative diseases. Prog. Neurobiol. 214:102270. doi: 10.1016/j.pneurobio.2022.102270 35447272

[ref26] Spotorno N. Najac C. Stomrud E. Mattsson-Carlgren N. Palmqvist S. van Westen D.. (2022). Astrocytic function is associated with both amyloid-β and tau pathology in non-demented APOE ϵ4 carriers. Brain Commun. 4, 1–9. doi: 10.1093/braincomms/fcac135, PMID: 35702728 PMC9185373

[ref28] van Eck N. J. Waltman L. (2017). Citation-based clustering of publications using CitNetExplorer and VOSviewer. Scientometrics 111, 1053–1070. doi: 10.1007/s11192-017-2300-7, PMID: 28490825 PMC5400793

[ref29] Wang H. Fu H. Fu Y. Jiang L. Wang L. Tong H.. (2022). Knowledge mapping concerning applications of nanocomposite hydrogels for drug delivery: a bibliometric and visualized study (2003-2022). Front. Bioeng. Biotechnol. 10:1099616. doi: 10.3389/fbioe.2022.1099616, PMID: 36686234 PMC9852897

[ref30] Wang C. Jing H. Sun Z. Yao J. Zhang X. Liu T.. (2021). A bibliometric analysis of primary aldosteronism research from 2000 to 2020. Front. Endocrinol. 12:665912. doi: 10.3389/fendo.2021.665912, PMID: 33986730 PMC8111213

[ref31] Werner P. Friedland R. P. Inzelberg R. (2015). Alzheimer's disease and the elderly in Israel: are we paying enough attention to the topic in the Arab population? Am. J. Alzheimers Dis. Other Dement. 30, 448–453. doi: 10.1177/1533317515577130, PMID: 25794510 PMC10852548

[ref33] Wu H. Zhou Y. Wang Y. Tong L. Wang F. Song S.. (2021). Current state and future directions of intranasal delivery route for central nervous system disorders: a scientometric and visualization analysis. Front. Pharmacol. 12:717192. doi: 10.3389/fphar.2021.717192, PMID: 34322030 PMC8311521

[ref34] Xiong X. James B. T. Boix C. A. Park Y. P. Galani K. Victor M. B.. (2023). Epigenomic dissection of Alzheimer's disease pinpoints causal variants and reveals epigenome erosion. Cell 186, 4422–4437.e21. doi: 10.1016/j.cell.2023.08.040, PMID: 37774680 PMC10782612

[ref35] Yang Y. Arseni D. Zhang W. Huang M. Lövestam S. Schweighauser M.. (2022). Cryo-EM structures of amyloid-β 42 filaments from human brains. Science 375, 167–172. doi: 10.1126/science.abm7285, PMID: 35025654 PMC7612234

[ref36] Yu Z. Ye J. Lu F. Shen M. (2022). Trends in research related to ophthalmic OCT imaging from 2011 to 2020: a bibliometric analysis. Front. Med. 9:820706. doi: 10.3389/fmed.2022.820706, PMID: 35572958 PMC9091450

[ref37] Zhang J. Wang Y. Zhang Y. Yao J. (2023). Genome-wide association study in Alzheimer's disease: a bibliometric and visualization analysis. Front. Aging Neurosci. 15:1290657. doi: 10.3389/fnagi.2023.1290657, PMID: 38094504 PMC10716290

[ref38] Zulfiqar S. Garg P. Nieweg K. (2019). Contribution of astrocytes to metabolic dysfunction in the Alzheimer's disease brain. Biol. Chem. 400, 1113–1127. doi: 10.1515/hsz-2019-0140 31188740

