# Supplementary material for: The role of astrocytes in Alzheimer’s disease: a bibliometric analysis
Source: Front Aging Neurosci. 2024 Nov 27;16:1481748. doi: 10.3389/fnagi.2024.1481748 (PMC11632101; doi:10.3389/fnagi.2024.1481748)

1. bibliometrix

R version 4.4.1 (2024-06-14 ucrt) -- "Race for Your Life"

Copyright (C) 2024 The R Foundation for Statistical Computing

Platform: x86_64-w64-mingw32/x64

R是自由软件，不附带任何担保。

在某些条件下你可以将其自由分发。

用'license()'或'licence()'来看分发的详细条件。

R是个合作计划，有许多人为之做出了贡献.

用'contributors()'来看合著者的详细情况

用'citation()'会告诉你如何在出版物中正确地引用R或R程序包。

用'demo()'来看一些示例程序，用'help()'来阅读在线帮助文件，或

用'help.start()'通过HTML浏览器来看帮助文件。

输入'q()'退出R.

[Workspace loaded from ~/.RData]

> install.packages("bibliometrix")

WARNING: Rtools is required to build R packages but is not currently installed. Please download and install the appropriate version of Rtools before proceeding:

https://cran.rstudio.com/bin/windows/Rtools/

试开URL’https://cran.rstudio.com/bin/windows/contrib/4.4/bibliometrix_4.3.0.zip'

Content type 'application/zip' length 2476322 bytes (2.4 MB)

downloaded 2.4 MB

程序包‘bibliometrix’打开成功，MD5和检查也通过

下载的二进制程序包在

C:\Users\Anxiaoqiong\AppData\Local\Temp\RtmpYTGG8z\downloaded_packages里

> library(bibliometrix)

Please note that our software is open source and available for use, distributed under the MIT license.

When it is used in a publication, we ask that authors properly cite the following reference:

Aria, M. & Cuccurullo, C. (2017) bibliometrix: An R-tool for comprehensive science mapping analysis,

Journal of Informetrics, 11(4), pp 959-975, Elsevier.

Failure to properly cite the software is considered a violation of the license.

For information and bug reports:

- Take a look at https://www.bibliometrix.org

- Send an email to info@bibliometrix.org

- Write a post on https://github.com/massimoaria/bibliometrix/issues

Help us to keep Bibliometrix and Biblioshiny free to download and use by contributing with a small donation to support our research team (https://bibliometrix.org/donate.html)

To start with the Biblioshiny app, please digit:

biblioshiny()

> biblioshiny()

载入需要的程序包：shiny

Listening on http://127.0.0.1:5756


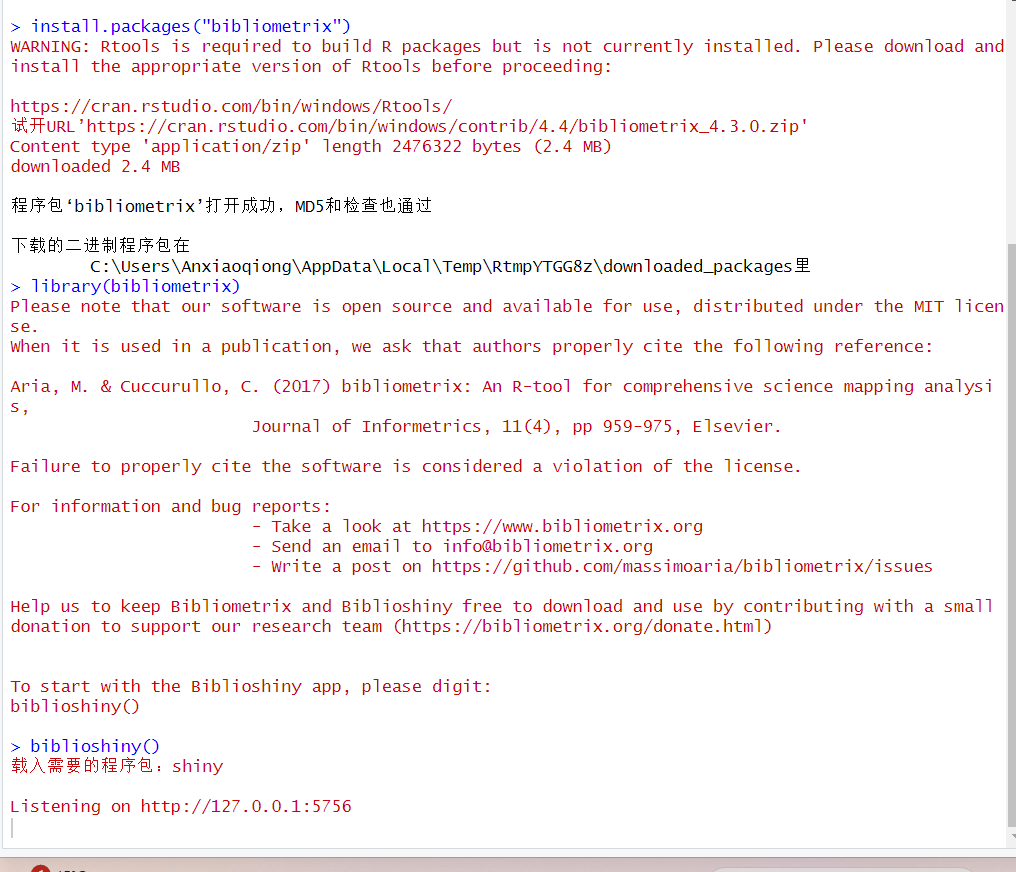


2 citespace

Networks(255): Time taken to merge networks: 0.018 seconds

Projects(737): The current project profiles are saved as C:\Users\Anxiaoqiong\citespace.projects.txt

Terms(201): You may create a file: C:\Users\Anxiaoqiong\.citespace\suffixes_to_retain.list

to provide a list of suffixes to retain. One suffix per line.

GraphPanel(4107): cardinality/(N*N)=0.56241876%


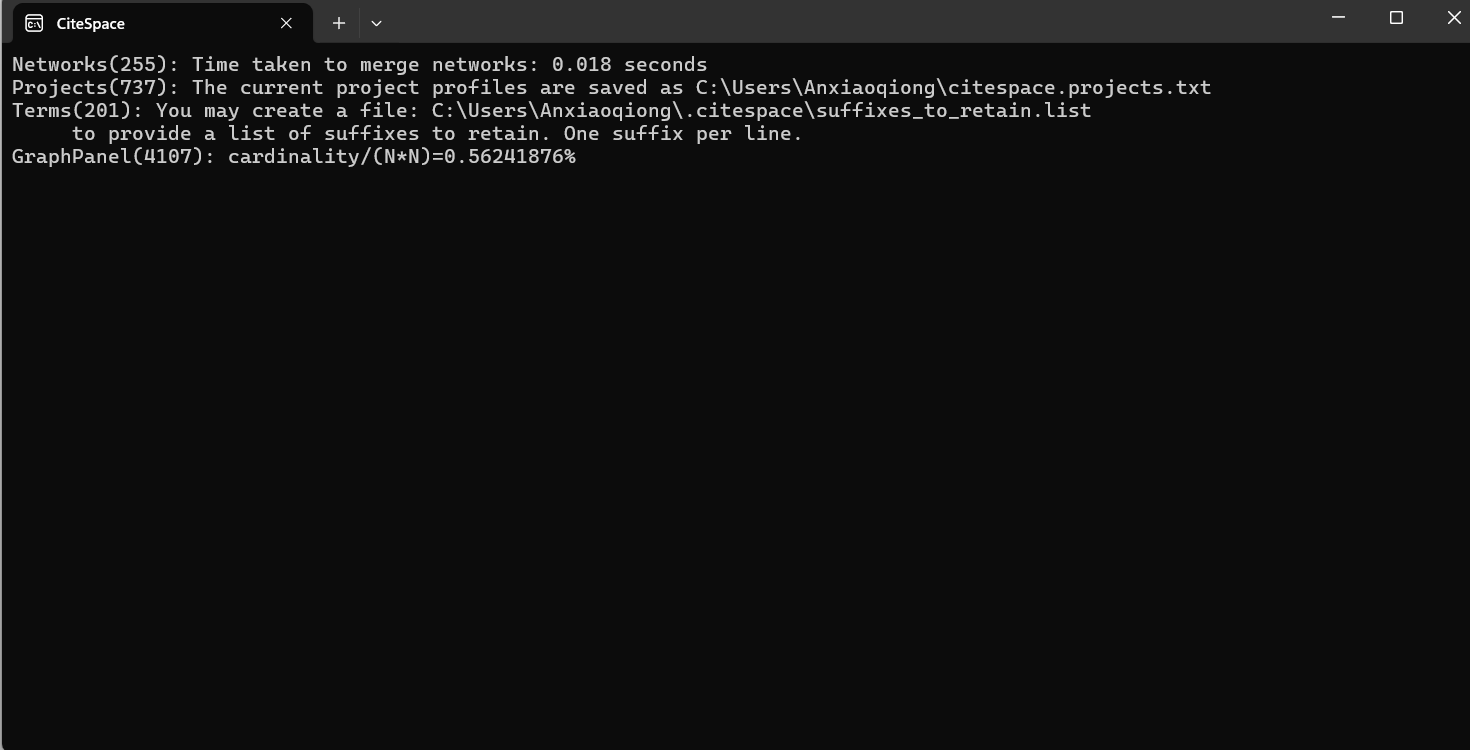

Supplement: Supplementary file 1 [file Table_1.DOCX]
